# P02.53. Examination of the association of diet on persistent cancer related fatigue

**DOI:** 10.1186/1472-6882-12-S1-P109

**Published:** 2012-06-12

**Authors:** S Zick, A Sen, T Han-Markey, R Harris

**Affiliations:** 1 University of Michigan, Ann Arbor, USA

## Purpose

Persistent cancer related fatigue (PCRF) is a common symptom experienced by many cancer survivors. While there is research recommending individualized nutritional counseling for improving fatigue during cancer treatment, there are no recommended dietary treatment options for PCRF.

## Methods

A cross-sectional study was conducted to examine possible associations between diet and fatigue. Dietary intake using a 4-day food diary was assessed in 40 cancer survivors. Cancer survivors had completed all cancer treatments at least 12 weeks prior and had their fatigue assessed with the Brief Fatigue Inventory (BFI). Participants were placed into one of three fatigue levels, “no fatigue”, “moderate fatigue”, or “severe fatigue” based on their BFI. Mean differences in food group, eating patterns and nutrient intake were analyzed using ANOVAs, K-means cluster analysis, chi-squares and proportional odds model.

## Results

Mean daily intake of whole grains, vegetables, and in particular green leafy vegetables and tomatoes, were significantly higher in the non-fatigued group compared to the moderately or severely fatigued cancer survivors. Also, cancer survivors reporting no-fatigue had significantly higher intakes of certain anti-inflammatory nutrients. Three different eating behaviors were identified: “Refined Carbohydrates”; “Healthy Eating”; and “Red Meat.” The probability of having severe fatigue versus the probability of having moderate or no fatigue was 1.28 greater in the “Refined Carbohydrates” pattern and 10.90 greater in the “Red Meat” pattern versus the “Healthy Eating” pattern. More people who were fatigued infrequently ate Ready-to-eat-breakfast-cereal (RTEBC) compared to non-fatigued cancer survivors and had a significantly worse overall nutrient profile.

## Conclusion

Increased consumption of RTEBC, whole grains and vegetables as well as foods rich in certain anti-inflammatory and anti-oxidant nutrients are associated with decreased levels of PCRF. Further rigorous studies will be required to investigate possible mechanisms and causal relationships regarding the benefits of particular nutrients, foods or diets on PCRF.

